# Essays on Hypochondriasis, and Other Nervous Affections

**Published:** 1816-11

**Authors:** 


### *Essays on Hypochondriasis, and other Nervous Affections.* By John Reid, M. D.

( Concluded from p. 226. )

We return with pleasure to this original and interesting performance, in which much science is diffused without making any ostentatious appearance; and many practical points of distinction are given without Didactic formality.

The 16th Essay on "Dyspeptic and Hepatic Diseases," comprehends many valuable rules for regimen, and several hints equally worthy of professional observation and private attention. We were particularly pleased with the following remark, because it exactly conveys our own sentiments, and shews that the zeal of improvement may sometimes, even in the best concerns, be carried beyond the bounds of discretion, and that regulations and institutions, which we may think evil or unnecessary, may be both salutary and advisable.

"The observance of fasts is a wholesome form of superstition. The omission of them in the Protestant calender, was, perhaps, as it relates to health, an unfortunate result of the reformation. Though no longer regarded by us as religious institutions, it would be desirable that some of them at least should be still kept with a kind of sacred punctuality, as salutary intervals of abstinence, which give to the stomach a periodical holiday, and afford an occasional respite from the daily drudgery of digestion." 142.

This Essay is properly followed by one on "Palsy, Idiocy, Spasmodic and Convulsive Affections;" the perusal of which will yield both instruction and entertainment. Among the cases here neatly reported and yet accurately described, we are particularly struck with one which we shall give in the author's words:

"About two years ago, I met with a remarkable case, which strikingly exemplified the connexion and affinity that may exist between what are called 'bilious affections,' and those which belong more apparently and decidedly to the nervous system. The patient referred to, had, in consequence of a severe domestic privation, been seduced into habits of intemperance, which, for two years, seemed to have no effect but upon the liver, producing at nearly regular intervals of ten days, vomitings of bile, occasionally accompanied by a diarrhœa, which, when combined with the former, of course assimilated the disease to the character of cholera. For the considerable period above mentioned, his only apparent complaint was what, in popular and fashionable language, is called the 'bile.' After the lapse, however, of somewhat more than two years from the commencement of his intemperate habits, without having received any precautionary or prefatory intimation, he was surprized by a seizure which paralyzed one half of his body, dividing it longitudinally into two equal sections, the one dead to all the purposes of sensation or voluntary motion, the other retaining the functions and privileges of vitality, although in some measure, of course, clogged and impeded by the impotent and diseased half to which it was united. When I saw him last, he had remained three years in this truly melancholy state. At least, during that time, he had experienced no important or permanent amelioration, nor any evident tendency towards the recovery of his corporeal powers. His mind also seemed to have shared in the paralysis. This was more particularly obvious in the lapses of his recollection. His memory had been maimed by the same blow which had disabled one side of his body. His recollection with regard to things, did not seem to be much impaired, but it was surprisingly so with regard to the denominations of persons or places. He has often forgotten the name of an intimate friend, at the very time that, with the most unaffected cordiality, he was shaking hands with him. Upon inquiry, it appeared that the pernicious habits of the patient were still persisted in; a circumstance which alone was sufficient to account for the uninterrupted continuance of his disorder.

" In this case, nothing could be more evident than that the bilious symptoms with which he was first affected, and the nervous complaints which succeeded, both originated from one source: and this may give a hint to those who are much troubled with the *bile*, as it is called, especially when it has been occasioned by the same means as in the instance just stated, that unless they seasonably reform their regimen, they may be at no great distance from a paralytic seizure." 158-162.

Dr. Reid speaks slightingly of the Bath waters as a remedy in paralytic cases, nor is he much more favourable to electricity; and, as he very properly observes,

" In the treatment of disease, it must appear desirable to effect the cure, when it is practicable, by means which act generally and impartially upon the body, rather than by those which operate, although not slowly, yet more immediately and with peculiar force, upon the delicate nerves and fibres of the stomach. The health, and of course comfort of man, depend, in a principal degree, upon the due vigour of his powers of digestion, which, by the inordinate or unnecessary use of drugs, has, in too many instances, been gradually impaired, and at length irrecoverably destroyed. This is apt to be the case, more especially with those fashionable hypochondriacs who are continually having recourse to the doses of pharmacy, in order to relieve the *ennui* of indolence, or to support the languor of an effeminate or enervated constitution. Such an existence as theirs may, out of courtesy, be called life, but it possesses none of life's privileges or its blessings." 176-177.

An ingenious, but rather brief Essay on "Hereditary Madness," is the next in succession; and we should have been glad to have seen so excellent a disquisition upon a subject of great importance to society more extended; and our readers, no doubt, will be of the same opinion, after perusing the following remarks on the duty of celibacy in those who are radically of a morbid intellect.

"Nothing can be more obvious, than that one who is aware of a decided bias in his own person towards mental derangement, ought to shun the chance of extending and of perpetuating, without any assignable limit, the ravages of so dreadful a calamity. No rites, however holy, can, under such circumstances, consecrate the conjugal union. In a case like this, marriage itself is a transgression of morality. A man who is so situated, in incurring the risk of becoming a parent, involves himself in a crime, which may not improbably project its lengthened shadow, a shadow too which widens, in proportion as it advances, over the intellect, and the happiness of an indefinite succession of beings." 185-186.

The 19th Essay, "on Old Age," contains some good moral observations on the desire of longevity; but, disposed as we are to admire the penetration and judgement of the author, even in metaphysics, we cannot assent to his assertion, that "an old man is no longer susceptible of new ideas;" and that "his mind lives altogether upon the past." So far, indeed, as this may be said of the general character of climacterics with respect to the study of new arts and languages, we are of the same opinion: but many instances might be adduced of men who have been too much devoted to pleasure or business to study in the prime of their years, have in the decline of life attained a competent degree of knowledge, and acquired a relish for inquiry which has been productive of the best consequences.

The Essay on "Lunatic Asylums" cannot fail to be read with avidity at a time like this, when these receptacles have, in an uncommon degree, excited the public attention and parliamentary investigation. This part of the work was written long before the subject had become a matter of general observation, and yet evils which have been developed by authority did not escape his examination, as appears from the following extract:

"A heavy responsibility presses upon those who preside or officiate in the asylums of lunacy. Little is it known how much injustice is committed, and how much useless and wantonly inflicted misery is endured in those infirmaries for disordered, or rather cemeteries for deceased intellect. Instead of trampling upon, we ought to cherish, and by the most delicate and anxious care, strive to nurse into a clearer and brighter flame the still glimmering embers of a nearly extinguished mind.

"It is by no means the object of these remarks to depreciate the value of institutions which, under a judicious and merciful superintendance, might be made essentially conducive to the protection of lunatics themselves, as well as to that of others, who would else be continually exposed to their violence and caprice. But it is to be feared, that many have been condemned to a state of insulation from all rational and sympathising intercourse, before the necessity has occurred for so severe a lot. Diseased members have been amputated from the trunk of society, before they have become so incurable or unsound as absolutely to require separation. Many of the depots for the captivity of intellectual invalids may be regarded only as nurseries for and manufactories of madness; magazines or reservoirs of lunacy, from which is issued, from time to time, a sufficient supply for perpetuating and extending this formidable disease,—a disease which is not to be remedied by stripes or strait-waistcoats, by imprisonment or impoverishment, but by an unwearied tenderness, and by an unceasing and anxious superintendance.

"The grand council of the country ought to be aroused to a critical and inquisitorial scrutiny into the arcana of our medical prisons, into our *slaughter-houses* for the destruction and mutilation of the human mind."

The 21st Essay is "On the Importance of counteracting the tendency to Mental Disease;" in which are these remarks upon one of the most important considerations arising out of the subject of insanity, as affecting the medical character in the reliance placed upon its testimony:

"Lucid intervals are a subject deserving of the very particular study of the legal, as well as the medical profession. There are, in fact, few cases of mania, or melancholy, where the light of reason does not now and then shine between the clouds. In fevers of the mind, as well as those of the body, there occur frequent intermissions. But the mere interruption of a disorder is not to be mistaken for its cure, or its ultimate conclusion. Little stress ought to be laid upon those occasional and uncertain disentanglements of intellect, in which the patient is for a time only extricated from the labyrinth of his morbid hallucinations. Madmen may shew, at starts, more sense than ordinary men. There is perhaps as much genius confined, as at large; and he who should court corruscations of talent, might be as likely to meet with them in a receptacle for lunatics, as in almost any other theatre of intellectual exhibition. But the flashes of wit betray too often the ruins of wisdom, and the mind which is conspicuous for the brilliancy, will frequently be found deficient in the steadiness of its lustre"

In the 22d Essay, the author treats of the use and abuse of "Bleeding;" which is admitted to be absolutely necessary in true pleurisy, but censured in strong terms, when indiscriminately resorted to in all cases of palsy and apoplexy.

On the subject of "Pharmacy," which is treated in the next Essay, the author very properly deprecates the practice of prolonging a medicinal course in cases of convalescence from acute disease; and the following observations, by the way of analogy, are equally deserving of the serious consideration of valetudinarians and practitioners.

"In the prescriptions of physicians, as well as in the preparations of cookery, a simplicity ought to be observed, which is in general, perhaps, not sufficiently attended to. A number of different dishes, which, separately taken, might be wholesome and nutritious, must altogether form a compound that cannot fail to have an unfavourable and disturbing effect upon the organs of digestion. In like manner, a glass of Port wine or a glass of Madeira, a draught of ale, or one of porter, might, in a state of debility or fatigue, for a time at least, invigorate and refresh; while if we take a draught, the same in quantity, but composed of all these different liquors, we shall find that, instead of enlivening and refreshing, it will nauseate and oppress. And yet something similar to this daily takes place in the formulæ of medical practitioners. A variety of drugs are often combined in the same recipe, each of which might be good, but the whole of which cannot. A mixture of corroborants or tonics, is not necessarily a tonic or corroborative mixture. A prescription ought seldom, perhaps, to contain more than one active and efficient ingredient; we should thus give that ingredient fair play, and by a competent repetition of trials might be able to ascertain, with tolerable correctness, its kind and degree of influence upon the constitution: whereas, out of a confused and heterogeneous mass, it is impossible for us to discriminate the individual operation of any one of the articles which compose it." P. 228-230.

In the 24th Essay, "Ablution" is considered in a similar manner with a decisive approbation of the use of cold water, as the means of preserving health, and of restoring it in particular diseases. But at the same time, the idea of superior advantages to be derived from sea-bathing is ridiculed with effect.

"Bodily exercise" is strongly recommended in the 25th Essay; in which, among many other acute remarks, we were particularly struck with the following:

"Improvements in the mechanism of modern carriages, by which they are made to convey a person from place to place, almost without giving him a sense of motion, may be one of the circumstances that have contributed to the increased prevalence of those maladies which originate in a great degree from a fashionable indulgence in lassitude and languor."

The next Essay has for its title, "Real Evils, a Remedy for those of the Imagination;" and, with the relation of some curious cases, it exhibits observations which may be considered as judicious hints for practice.

The last Essay in the volume is on the advantages arising from "Occupation;" the necessity of which is enforced by solid reasoning, apt illustration, and a singular case, with which we shall conclude our extracts, already sufficiently numerous.

"I was once consulted by a hypochondriacal patient, who had been the greatest part of his life a journeyman taylor, but who, by an unexpected accident, became unhappily rich, and consequently no longer dependent for his bread upon drudgery and confinement. He accordingly descended from his board: but Charles the Fifth, after having voluntarily descended from his throne, could not have regretted more severely the injudicious renunciation of his empire. This man, after having thrown himself out of employment, fell ill of the tedium of indolence. He discovered, that having nothing to do, was more uncongenial to his constitution, even than the constrained attitude, and the close and heated atmosphere in which he had been accustomed to carry on his daily operations. In one respect, however, the repentant mechanic was less unfortunate than the imperial penitent. It remained in the power of the former to reinstate himself in his former situation; which, after having resumed it, no motive could, a second time, induce him to relinquish." P. 260-262.

After so copious a view and minute an analysis of the present volume, any thing farther that we could say concerning it must be needless; the reader will see by the subjects treated, that the work is one of universal interest, because there is no human being, capable of thinking, who has not his seasons of mental depression or excessive irritation, who is not either called upon to watch over his own infirmities, or to commiserate those of others. In the extensive range of moral and actual ills, there is not one that is so frequently obtruded upon our feelings as nervous sensibility; and, therefore, a more benevolent office can hardly be undertaken, than that of pointing out the varieties of this Protean malady, and the causes which tend to its ascendancy over the body and mind, till the grave closes upon the one, or reason is extinguished in the other.

